# Gambling-Related Harms for Affected Others: A Finnish Population-Based Survey

**DOI:** 10.3390/ijerph18189564

**Published:** 2021-09-10

**Authors:** Sari Castrén, Kalle Lind, Heli Hagfors, Anne H. Salonen

**Affiliations:** 1 Health and Well-Being Promotion Unit, Finnish Institute for Health and Welfare, P.O. Box 30, 00271 Helsinki, Finland; kalle.lind@thl.fi (K.L.); anne.salonen@thl.fi (A.H.S.); 2 Social Sciences Department of Psychology and Speech-Language Pathology, University of Turku, 20014 Turku, Finland; 3 Department of Medicine, University of Helsinki, P.O. Box 64, 00013 Helsinki, Finland; 4 Faculty of Social Sciences (SOC), Tampere University, 33014 Tampere, Finland; heli.hagfors@tuni.fi; 5 Faculty of Health Sciences, University of Eastern Finland, P.O. Box 1627, 70211 Kuopio, Finland

**Keywords:** affected others, affected family members, affected non-family members, problem gambling, gambling-related harm, population study

## Abstract

Aims This study explores the prevalence of being a past-year affected other (AO) of a problem gambler by gender. The aims were to study the amount and type of gambling-related harms (GRHs) for subgroups of AOs and to distinguish GRH profiles for AO subgroups. Methods A total of 7186 adults aged 18 years and over participated in the Gambling Harms Survey evaluating year 2016. The data were analyzed using descriptive statistics and binary logistic regression. Results Of all respondents, 12.9% were defined as past-year AOs (women 13.7%; men 12.1%). The proportion of affected non-family members (ANFs) was 8.4%, and 5.6% were affected family members (AFMs). AFMs were usually women, and ANFs were usually men. Emotional, relationship, and financial harms were the most common types of harm. The odds of experiencing financial harm were highest for the 18- to 34-year-olds (OR 1.82) and for those whose partner/ex-partner had a gambling problem (OR 3.91). Having a parent/step-parent (OR 1.93) and child/stepchild (OR 3.64) increased the odds of experiencing emotional harm, whereas male gender (OR 0.50) and being an ANF (OR 0.58) decreased emotional harm. Relationship harm was evident for partners/ex-partners (OR 1.97–5.07). Conclusions GRH profiles for AO subgroups varied, which emphasizes the need for effective harm minimization strategies for those in need.

## 1. Introduction

The recent research scope has been broadened to look at different types of gambling-related harms and identify who is encountering these harms [1,2]. Based on the framework by Langham and her colleagues (2016), gambling-related harms (GRHs) are defined as any negative/detrimental consequence caused by gambling that leads to diminished health or well-being of an individual, family unit, community, or population. This is particularly true among persons close to the problem gambler [3,4,5]. Thus, the need for support and treatment for close ones has been acknowledged [5,6,7,8,9], and a profound understanding of GRHs of close ones and subtypes are needed. This study investigates GRHs exclusively based on the experiences of different subgroups of affected others with a close relationship to a problem gambler. According to Langham and colleagues, GRHs include financial harms, relationship harms, and emotional/psychological harms; the impacts on health, work, and study; and social deviance harms, such as child neglect and possible criminal acts [1]. Overall, financial, emotional, and relationship harms have been identified as the most prevalent types of GRHs for affected others [5,10,11]. GRHs for the affected others are largely similar to those of gamblers [10,12]. At the same time, population-based studies of GRHs among affected others are relatively scarce.

### 1.1. Prevalence of Affected Others in Different Subgroups

The term affected other (AO) is used to refer to persons who have or have had close ones with problematic gambling. The term AO does not imply relationship status or any level of concern [13]. AO is used as a synonym for the concerned significant other (CSO) of a person with a gambling problem [14,15,16]. Herein, the subgroups of AOs are examined from the perspective of both affected family members and affected non-family members, including friends, co-workers, neighbors, and other close persons.

The prevalence of AOs varies, drawn from epidemiological studies from 2 to 19 percent, depending on methodology, timeframe, and definitions used [14,15,16,17,18,19]. In Finland, the prevalence of AOs has increased in the past years from 13% to 19% [19,20]. This is a substantial number of individuals in the population that ought to be recognized. Typically, women are affected family members and men are affected close friends [14], with the most affected age groups being 18–24 and 25–44 [15,16]. GRHs for different subgroups of AOs, may vary.

### 1.2. GRHs for Affected Family Members

The link between health issues, including a gambling problem of one’s own, poor perceived health, psychological distress, and risky alcohol consumption and being an AO was clearest among the affected family members (AFMs) [5,14,15,16,17,18]. Likewise, with the health correlates, the amount of GRHs was the largest and most extensive among the AFMs. Particularly partners report engaging in dysfunctional behaviors such as risky alcohol consumption, smoking, overeating, and compulsive buying to cope with the distress [8].

Problem gambling affects the family as a whole. Gamblers’ partners report severe emotional and psychological harms, such as feelings of extreme distress, hopelessness, and vulnerability, as well as stress-related health harms including insomnia, headaches, and high blood pressure [8,10,12,21]. Some spouses report getting extra employment or paying their partner’s debts to overcome financial difficulties [15,21]. This can create tension in relationships that may manifest in arguments, mistrust, the threat of separation or divorce [16,21], and even risk of experiencing familial violence [22].

Gambler’s children have an elevated risk for developing gambling problems themselves and engaging in other risky behaviors such as alcohol consumption, smoking, and substance abuse [23]. Gambling in a family affects a child’s academic performance and may contribute to difficulties in school, such as behavioral and adjustment problems [8]. Gambling may affect and change the family dynamics: the gambling parent might neglect their children by leaving them unattended while gambling [8,10], or a shift in family responsibility may occur as children take control of the family’s financial situation and the role of a caregiver, or distance themselves from a gambling parent [1,8]. Only a few studies have explored how an adult child’s problem gambling affects parents [24,25].

Parents and grandparents of a problem gambler report diminished enjoyment of life, physical and emotional stress-related symptoms, financial harms, and relationship harms. Trying to help by lending money or paying the gambler’s debts or dealing with the loss of money or sold household items is common and causes mixed harms to AOs.

### 1.3. GRHs for Affected Non-Family Members

Although AFMs experience GRHs more often and to a greater degree than an affected non-family member (ANF), ANFs still experience a substantial amount of GRHs. According to previous studies, the person experiencing GRH is most often a close friend of a problem gambler [18,26], yet very little is known about how problem gambling affects friends of a gambler. However, one’s own gambling participation as well as one’s own gambling problem and risky alcohol consumption are linked with being a friend of the person with a problem gambling [19].

GRHs at the workplace are identified by numerous studies, which conclude that gambling at work means that the worker is not fulfilling their part of the employment agreement. Gambling even during leisure time may affect work performance (inefficient work), absenteeism, and tardiness at work—being preoccupied with gambling at work and experiencing withdrawal symptoms or asking for loans from co-workers [26,27,28,29,30].

Previous research concerning GRHs of AOs has focused mainly on the problem gambler’s family, especially the partner’s experiences, while little is known from the perspective of ANFs. Besides, ANFs (namely friends) have been recognized better than other non-family members. Our aim is to explore the prevalence of being an AO of a problem gambler by gender, and the amount and type of GRHs for subgroups of AOs and to characterize unique GRH profiles for AO subgroups.

## 2. Methods

### 2.1. Data Collection

In this study, we use the first wave of data from the population-based Finnish Gambling Harms Survey [19,30] conducted by the Finnish Institute for Health and Welfare. The data were collected by Statistics Finland and portrays the situation in three Finnish regions (Uusimaa, Pirkanmaa, and Kymenlaakso) during the year 2016.

The data were collected between January and March 2017 from adults aged 18 years and over. Both web and postal surveys were available in both official languages, Finnish and Swedish. A total of 20,000 potential participants were randomly selected from the population information system and were invited to the study with an invitation letter and a brochure. Oversampling 18 to 24-year-olds compensated for the low participation rate expected based on previous surveys [20]. Persons living in institutions, such as prisoners and the infirm, were excluded. Furthermore, non-eligible individuals (*n* = 67) were removed from the sample. Therefore, the final sample consisted of 19,933 persons. The final response rate was 36.1% since 7186 adults participated in the survey.

Of the respondents, 71% (*n* = 5084) participated using the online survey, whereas 29% (*n* = 2102) participated through the postal survey [31]. Women and older respondents were more active in taking part, whereas men and younger respondents were more reluctant to participate [19]. Ultimately, almost half (48%) of the respondents were men, and the average age of respondents was 49 years (*SD* = 18.4) [19].

### 2.2. Affected Others

Affected others (AOs) were evaluated by inquiring: “During the year 2016, has there been a person in your life that you consider gambles too much?” [31,32]. If the person responded affirmatively, the following question was asked: “What is this person’s relationship to you?” Ten options for AOs were available: (1) Spouse/partner, (2) parent/step-parent, (3) child/stepchild, (4) other person (in your household), (5) other family member (not in your household), (6) ex-partner, (7) work colleague, (8) friend, (9) neighbor, and (10) other person. First, three variables were created to indicate whether the respondent was an AO (options 1−10), AFM (options 1−3 or 5–6), or ANF (options 4 or 7−10). In addition, single response options were recoded due to the small number of respondents in some subcategories (Table 1Table 2 and Table 3. No results were reported if the frequency was less than 5.

**Figure 1:**
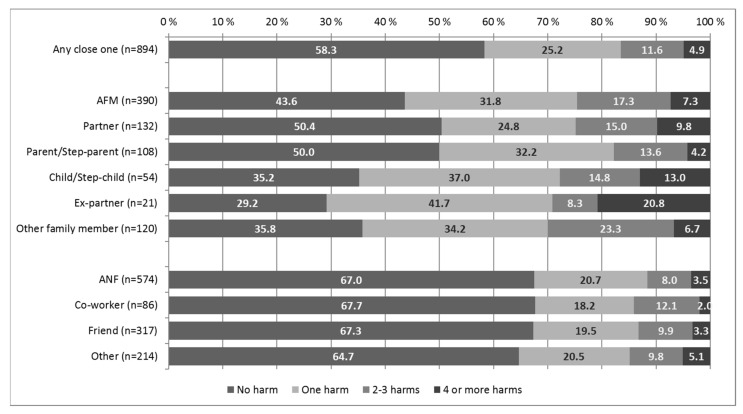
The amount of gambling harms for the AOs (n) by the problem gambler’s relationship to the AO.

GRHs for AOs were questioned using 12 response options [18]. In addition to an open-ended response option, a question about work/study-related harm was added. Furthermore, a new variable indicating the amount of GRHs was recoded (Figure 1). Harm categories and the individual items described the type of GRHs.

In addition, the data contained register-based information about the respondents’ gender and age. Age was dichotomized into two age groups: 18−34, and 35 and up.

### 2.3. Data Analyses

The age, gender, and regional distributions were used to calibrate the data weights. Percentages and significance (*p*) were calculated with Chi-Squared and Fisher’s exact tests. All selected variables were dichotomized and added simultaneously into the logistic regression models. Furthermore, health-related harms, work/study harms, and social deviance harms were combined for the model and named other harms due to the small number of respondents in some subcategories. To optimize the model, gender and age were added. All analyses were done using SPSS version 27.0 (IBM Corporation, Chicago, IL, USA).

## 3. Results

### 3.1. The Proportion of AOs and the Problem Gambler’s Relationship to the AO

Of all respondents, 12.9% had at least one person in 2016 who they considered a problem gambler (Table 1). The proportion of AOs was significantly higher among women (13.7%) than men (12.1%). The proportion of past-year ANFs was 8.4%, while the corresponding figure for the AFMs was 5.6%. Overall, being an AFM was more common (7.4%) among women than among men (3.6%), whereas being an ANF was more common among men (9.2%) than among women (7.7%). One percent of all respondents were both AFMs and ANFs.

Among AFMs, the problem gambler was most commonly a partner or ex-partner (1.9%) or a parent or step-parent (1.7%). Among ANFs, the problem gambler was most commonly a friend (4.6%) or a co-worker (1.4%). The proportion of those with a friend and co-worker with a gambling problem was higher among men.

### 3.2. The Amount of GRHs for the AOs

More than half (58.3%) of the AOs did not experience any harm (Figure 1). Among AFMs, the proportion of those experiencing no harm was 43.6%, while the corresponding proportion among ANFs was 67.0%. Four or more harms were experienced most frequently by the AFMs (7.3%). Among AFMs, those with a child or stepchild (13.0%) or partner (9.8%), and particularly an ex-partner (20.8%) most often experienced at least four harms. Among ANFs, the proportion of those experiencing four or more harms varied between 2.0% and 5.1%.

### 3.3. The Type of GRH by Harm Category

Emotional harms, relationship harms, and financial harms were the three most common types of past-year harms for AOs—both AFMs and ANFs (Table 2). Among the AOs, the most common individual harms caused by close ones’ problem gambling were worry about the health or well-being of other close ones (16.5%), emotional distress (14.7%), and other interpersonal relationship problems, such as arguments, isolation, and distancing from a friend (8.8%). Furthermore, undefined harms, such as loss of time and money, undefined feelings of pity and grief, worry, or self-destructive thoughts were experienced by one in ten (9.1%). Overall, the proportions of harm for AFMs were mostly higher than those of ANFs, except for work- and study-related harms. Among AFMs, 1.3% had experienced such harms while the corresponding proportion among ANFs was 2.7%.

Of family members, the highest prevalence of experiencing at least one harm type was among those with ex-partners (70.8%), a child or stepchild (64.8%), or other family members (64.2%) with a gambling problem (Table 3). The proportion of those experiencing at least one financial harm was highest among those with an ex-partner with a gambling problem (26.1%). On the other hand, the proportion of those experiencing at least one emotional harm was highest among those having a child or stepchild with a gambling problem (60.0%), while relationship harms were most common with partners and ex-partners. Of non-family members, about one in three (32.9−35.3%) had experienced at least one harm—most commonly emotional harms.

The odds of experiencing financial harms were significantly increased for 18 to 34-year-olds (OR 1.82) and for those whose partner or ex-partner had a gambling problem (OR 3.91) (Table 4). Furthermore, having a parent or step-parent (OR 1.93) and child or stepchild (OR 3.64) increased the odds of experiencing emotional harm, whereas male gender (OR 0.50) and being an ANF (OR 0.58) decreased the odds of experiencing emotional harm. Those whose partner (OR 5.07) or ex-partner (OR 1.97) had a gambling problem had increased odds of experiencing relationship harm. Moreover, the odds of experiencing other harms—either social deviance harm, work- or study-related harm, or health harm—were increased if one had a partner (OR 3.31) or ex-partner (OR 2.99) with a gambling problem.

## 4. Discussion

This study explored the prevalence of being an AO of a problem gambler by gender. In addition, it focused on the amount and type of GRHs for AOs, including both AFMs and ANFs, and distinguished the GRH profiles for AO subgroups. A past-year time frame was used; therefore, this study differs from previous Finnish studies on the same topic, which were conducted using a lifetime frame [11,14,19].

As expected, the AFMs were typically women. However, ANFs (including both friends and co-workers) were typically men. Similar gender differences have been seen among both the general population [14,19,21,32] and in treatment settings. In the latter context, women AOs—especially affected female partners and mothers—have been extensively represented [33,34,35,36,37,38,39]. ANFs were typically men, thus rarely observed as seeking help for themselves as AOs. Help-seeking behavior is plausible when further exploring the type and prevalence of harms experienced by AFMs and ANFs.

Our results confirmed that emotional harms, relationship harms, and financial harms were the three most common types of past-year harms for the AOs [5,10,11]. Different types of harms may be interconnected and boost each other. For example, a financial burden may intensify relationship and emotional harms. The above-mentioned harms were among the top three with both the AFMs and the ANFs; but in general, the ANFs experienced fewer harms than the AFMs. This finding makes sense since the nature of the relationship between the ANF and the problem gambler is generally assumed to be more distant than the relationship with the AFM, at least considering finances.

Emotional harms were experienced largely by AFMs (43.1%) and less than half by ANFs (21.1%). Based on previous research, affected partners report that gambling impacts their daily life and brings distress, a desire to escape the relationship, a sense of hopelessness, and helplessness [12]. It is obvious that the worry and stress of a problem gambler’s overall life course, including possible criminal acts and the financial situation, impose an emotional burden and affect one’s own well-being.

In our study, emotional harms were most common and enhanced if the child or stepchild or partner or ex-partner was the problem gambler. This implies that emotional harm may be perceived differently, depending on the type and depth of the relationship but also depending on the distance from the gambler. For example, a person living in the same household with a problem gambler may experience distress to handle the household finances and daily errands because the gambling problem may limit a person’s ability to commit and share duties equally. Additionally, family members living with a problem gambler may have to constantly face the GRHs without any possibility to withdraw.

Affected partners and also ex-partners came across various harms rather commonly. Based on our model, relationship and financial harms were exceptionally frequent among the affected partners or ex-partners. Relationship harms have been reported by partners as being five to six times higher than the gambler’s perception of the state of the relationship [20]. These differences may be affected by the gambler’s preoccupation with gambling, which manifests in lower interest in relationships in general [40]. The severity of this domain of harm should be acknowledged, particularly with partners, since it may and often does lead to the end of the relationship [12,41].

Around one in ten AOs had experienced financial harms. Being an affected partner often means taking responsibility for running the household finances [42]. In some cases, it even means taking on extra employment, paying the partner’s debts to overcome financial difficulties [15,21] or facing indebtedness and creditor issues [1]. Being an affected partner can influence work performance, and prolonged distress may lead to poor mental health and risky alcohol consumption [15,21]. Financial harms, such as loans or the consequences of bankruptcy may even follow along long after a separation or divorce. The continuation of the gambler’s financial struggle may also negatively affect the ex-partners’ and mutual children’s long-term quality of life with delays in child support payments or child neglect.

On the other hand, gambling and problem gambling can occur anywhere, even in the workplace. The proportions of harms for ANFs were mostly lower than those of AFMs, but work- and study-related harms were more common among the ANFs. In fact, there are some specific work fields—such as the transport sector, shift work, building, construction and service, monotonous manual indoor work, and work that requires frequent traveling [27,28,30] where the prevalence of gambling at work is higher than average. Above all, employees working at gambling venues have high rates of gambling participation and problem gambling [43]. Exposure to gambling at work, and also in connection to work (i.e., close proximity to gambling venues), may expose one to gambling at lunch breaks, for example [30]. Gambling can also be a part of the workplace culture and, in this way, it can fuel an existing gambling problem [27]. Overall, the harms for the ANFs were not as significant as the harms for the AFMs. Yet, it should not be overlooked. For a friend, it may mean constant worry about the gambler’s overall well-being, the disappointment of neglecting a friendship, and the strain that problem gambling causes for the gambler.

### 4.1. Implications for Practices and Further Studies

As per our results, GRHs vary in different subgroups. The first point is to increase public and professionals’ awareness of the gambling phenomena (i.e., problem gambling can cause harms to people close to the gambler) and to lower the bar of directly asking whether such a problem exists. The availability of low-threshold support or treatment options for AOs is still non-existent or limited, at least in Finland. Not all AOs report GRHs, and in turn, we do not know well enough who are the AOs that require support and what type of support; thus, this is an area worth investigating in the future. It could well be that the psychoeducational components of problem gambling (understanding a gambler), securing one’s own finances, and building clear boundaries to secure one’s own well-being delivered online as self-help [34,39,44] may be a suitable support for this subgroup. Overall, AOs may use self-help strategies that focus on changing their own behavior, particularly taking responsibility for the family’s finances [3]. Moreover, AOs may use strategies to support their close ones to change their gambling behavior by telling them how their gambling has impacted the family, for example. These self-help strategies can be matched with other support and treatments.

Furthermore, recognizing gambling in the workplace can be challenging since people are often not aware of the magnitude of gambling problems or even that the person close to them is gambling [25] due to its hidden nature. Clear guidelines of how an affected friend, co-worker, or family member can recognize and support the person with a gambling problem already exist [45] and can be used as guidelines in community training targeted to the wider public [46]. In a workplace, clear guidelines are recommended to be followed the same as workplace guidelines for a worker being at the workplace under the influence of alcohol or other drugs. Preventive and harm minimization strategies at the workplace, especially those workplaces that are at greater risk [27], should be put in place. Yet, we know little about how an adult child’s gambling affects the parents’ well-being, and that would be an important subgroup to explore more in depth.

It would be useful for policy makers to understand to what extent emotional, financial, and health harms are more widespread and prevalent than the ones experienced solely by the gamblers themselves. In fact, it has been estimated that problem gambling affects approximately six persons per gambler [13].

### 4.2. Limitations

In this study, only the personal views of AOs of problem gamblers were recorded, and no validated instrument was used. The prevalence rates of AOs were assessed using self-assessment method and a life-time frame. Even though it would be interesting to compare the prevalence of AOs along with the prevalence rate of problem gambling from the gamblers perspective, the comparison is not advisable due to the methodological differences. For example, the prevalence rate of problem gambling has been around 3 percent in Finland, but the South Oaks Gambling Screen was used with a past-year time frame. Furthermore, many subgroups of respondents and subcategories of GRHs were combined for the models due to low frequencies. Overall, the results are presented only if the frequency in the subcategory was above five, and this may have meant missing some detailed information. Additionally, non-significant findings may be explained by the small number of participants in some subgroups, even though the corresponding OR suggests an association between the variables. In our study, respondents could choose multiple options for the question about the respondent’s relation to the person with a gambling problem. Therefore, although the same question was used on our respondents, the results are not directly comparable with the results of the previous two studies where respondents were advised to choose only one option [31,32].

## 5. Conclusions

The AFMs were typically women, and ANFs (including both friends and co-workers) were typically men. Emotional harms, relationship harms, and financial harms were the three most common types of past-year harms for the AOs. Affected partners experienced various harms and rather commonly. Relationship and financial harms were exceptionally typical among both affected partners and ex-partners. Emotional harms were utmost distinctive. The most common and notable emotional harms were if the child or stepchild or partner/ex-partner was the problem gambler. About one in ten AOs had experienced financial harms. Even though the proportions of harms for ANFs were mainly lower than those of AFMs, the work- and study-related harms were more common among the ANFs. Given the significant proportion of AOs and the variety of GRHs they encounter regardless of subgroups, there is a need to implement preventive efforts, community training, and tailored intervention to minimize the harms of AOs.

## Figures and Tables

**Table 1:** The proportion of affected others (AOs) in 2016 and the problem gambler’s relationship to the AO by gender.

	All Respondents (*n* = 7186)		Females (*n* = 3760)		Males (*n* = 3426)		
	*n*	%	*n*	%	*n*	%	*p*
AO	894	12.9	522	13.7	372	12.1	0.046
AFM	390	5.6	277	7.4	113	3.6	≤0.001
Partner or ex-partner	132	1.9	94	2.5	38	1.2	≤0.001
Parent or step-parent	108	1.7	75	2.1	33	1.1	≤0.001
Child or stepchild	54	0.8	41	1.1	13	0.4	≤0.001
Other family member	120	1.7	85	2.2	35	1.1	≤0.001
ANF	574	8.4	294	7.7	280	9.2	0.019
Friend	317	4.6	145	3.7	172	5.7	≤0.001
Co-worker	86	1.4	28	0.8	58	2.0	≤0.001
Other ^a^	215	3.0	128	3.4	87	2.5	0.032

The proportion of affected others (AOs) in 2016 and the problem gambler’s relationship to the AO by gender. AO = affected other; AFM = affected family member; ANF = affected non-family member. The data (*n* = 7186; non-weighted) were weighted based on gender, age, and region of residence. Significance (*p*) was determined using Fisher’s exact test. ^a^ The category includes neighbors, other non-family members living in the same household, and all undefined persons. Note: it is possible that the respondents have several problem gamblers in their life, both in the family and outside the family.

**Table 2:** The type of gambling-related harms in 2016 by harm category among affected others (AOs).

Harm Type		AOs		AFMs		ANFs	
	Has a Gambling Problem of Your Close One(s) Caused You:	*n* = 894	%	*n* = 390	%	*n* = 574	%
Any harm		378	41.7	220	56.5	192	33.0
Emotional harm	Any emotional harm	261	28.7	167	43.1	118	20.2
	Worry about the health or well-being of other close ones	150	16.5	87	22.8	81	13.6
	Emotional distress, such as stress, restlessness, anxiety, depression, hopelessness, or guilt	135	14.7	98	24.8	51	8.8
	Worry about health or well-being of your own child	47	4.9	38	9.3	13	2.2
Relationship harm	Any relationship harm	118	12.9	74	18.8	59	10.3
	Problems in a relationship, such as arguments, distrust, divorce, or separation	60	6.4	42	10.5	22	3.5
	Other interpersonal relationship problems, such as arguments, isolation, distancing yourself from friends	78	8.8	45	11.8	45	8.0
Financial harm	Any financial harm	73	8.4	37	10.3	44	7.6
	Eviction or a threat of being evicted	16	1.8	7	1.8	11	2.0
	Other financial problems, such as payment issues, loans related to gambling, loss of financial credibility	69	7.9	35	9.8	42	7.3
Social deviance harm	Any social deviance harm	16	1.8	7	1.8	10	1.8
	Emotional violence, such as blackmailing, pressuring, and intimidation	12	1.3	6	1.5	7	1.3
	Physical violence witnessed or being threatened	10	1.2	..	..	..	..
	Victim of some other type of crime, for example, theft or identity theft	9	0.9	5	1.3	5	0.5
Health harm	Health impacts, such as sleep problem, headaches, backaches, or stomach aches	42	4.7	31	8.0	18	3.0
Work/study harm	Work- or study-related harm	18	2.1	5	1.3	14	2.7
Undefined harm	Other harm, please specify ^a^	84	9.1	41	10.0	47	8.0

AO = affected other; AFM = affected family member; ANF = affected non-family member. The AOs in the data (*n* = 894, non-weighted) were weighted based on gender, age, and region of residence. ^a^ Undefined harms included loss of time and money, undefined feelings of pity and grief, worry, and self-destructive thoughts. Note: it is possible that the respondents have several different problem gamblers in their life, both in the family and outside the family; Data not available or too uncertain for presentation or subject to secrecy.

**Table 3:** The type of gambling harms in 2016 for the AOs by the problem gambler’s relationship to the AO.

	Affected Family Members (AFMs) (*n* = 390)					Affected Non-Family Members (ANFs) (*n* = 574)		
Has a Gambling Problem of Your Close One(s) Caused You:	Partner	Ex-Partner	Parent or Step-Parent	Child or Stepchild	Other Family Member	Friend	Co-Worker	Other *
	*n* = 132	*n* = 21	*n* = 108	*n* = 54	*n* = 120	*n* = 317	*n* = 86	*n* = 214
1. Financial harm	14.4	26.1	5.9	11.1	10.0	7.5	9.1	8.4
2. Emotional harm	32.3	45.8	37.3	60.0	51.7	21.5	14.1	21.9
3. Relationship harm	29.3	26.1	11.9	11.1	20.0	10.8	11.1	13.5
4. Health harm	10.5	..	5.9	11.1	8.3	3.3	..	3.7
5. Work/study harm	..	..	..	..	..	1.5	6.1	4.2
6. Social deviance harm	..	..	..	..	..	1.5	..	..
Any harm	49.6	70.8	50.0	64.8	64.2	32.9	33.0	35.3

The numbers are percentages. AO = affected other. The AOs in the data (*n* = 894, non-weighted) were weighted based on gender, age, and region of residence. * The category includes neighbors, other persons living in the same household, and other undefined persons. Note: it is possible that the respondents have several different problem gamblers in their life, both in the family and outside the family; Data not available or too uncertain for presentation, or subject to secrecy.

**Table 4:** Logistic regression models on the association between different harm categories and relationship to the AO.

	Financial Harms		Emotional Harms		Relationship Harms		Other Harms ^a^	
	OR	95% CI	OR	95% CI	OR	95% CI	OR	95% CI
Male (ref. female)	1.31	0.80–2.15	0.50 ***	0.36–0.69	0.77	0.50–1.18	1.10	0.62–1.94
18 to 34-year-old (*ref. 35–74)*	1.82 **	1.11–2.99	1.35	0.99–1.86	1.50	0.99–2.25	1.33	0.75–2.36
AFM								
Partner or ex-partner	3.91 ***	1.95–7.83	1.03	0.61–1.75	5.07 ***	2.78–9.24	3.31 **	1.58–6.97
Parents or step-parent or other family member	1.51	0.77–2.95	1.93 **	1.19–3.10	1.97 *	1.12–3.46	1.75	0.85–3.61
Child or stepchild	2.29	0.87–6.02	3.64 ***	1.84–7.19	0.98	0.38–2.56	2.99 *	1.14–7.82
ANF	1.35	0.68–2.67	0.55 **	0.34–0.89	1.32	0.74–2.33	1.20	0.58–2.51
Log-likelihood	510.5		1008.9		669.1		421.6	
LR Chi 2	22.6		106.1		46.6		16.3	
*p*	0.001		0.000		0.000		0.012	
Nagelkerke R	0.055		0.154		0.091		0.046	

AO = affected other. AFM = affected family member. ANF = affected non-family member. The AOs in the data (*n* = 894, non-weighted) were weighted based on gender, age, and region of residence. ^a^ Includes social deviance harm, work, or study-related harm and health harm. * *p* < 0.05; ** *p* < 0.01; *** *p* < 0.001.

## Data Availability

The survey data without any register-based information is publicly accessible for research purposes from the Finnish Society Science Data Archive (FSD) with the name of Rahapelikysely 2016 (FSD3261), urn:nbn:fi:fsd:T-FSD3261.
